# Speed-sintering and the mechanical properties of 3–5 mol% Y_2_O_3_-stabilized zirconias

**DOI:** 10.1007/s10266-023-00796-y

**Published:** 2023-03-02

**Authors:** Julia Lubauer, Fernanda Haverroth Schuenemann, Renan Belli, Ulrich Lohbauer

**Affiliations:** Zahnklinik 1–Zahnerhaltung und Parodontologie, Forschungslabor für dentale Biomaterialien, Friedrich-Alexander-Universität Erlangen-Nürnberg (FAU), Glueckstraße 11, 91054 Erlangen, Germany

**Keywords:** Flexural strength, Yttria-stabilized tetragonal zirconia, Mechanical tests

## Abstract

Ever faster workflows for the fabrication of all-ceramic restorations are of high economic interest. For that purpose, sintering protocols have been optimized for use in modern sintering furnaces, the so-called speed-sintering. However, conventional furnaces are still the most widely used equipment to sinter zirconia restorations. In this in-vitro study, we evaluated the feasibility of a speed-sintering protocol using a conventional sintering furnace to sinter different dental zirconias (stabilized with 3 mol% up to 5.4 mol% Y_2_O_3_) in comparison to a conventional sintering program. The properties evaluated were Young’s modulus, Poisson’s ratio, density, biaxial flexural strength, and fracture toughness. We show here that despite differences being dependent on material, the physical and mechanical properties of speed-sintered zirconia are comparable to those obtained by the conventional sintering.

## Introduction

The clinical success of classical, conventionally sintered zirconia, namely, 3 mol% Y_2_O_3_-stabilized zirconia (3YSZ) is striking, as per the clinical evidence showing low fracture rates compared to glass–ceramics [1, 2]. The excellent clinical performance of 3YSZ is credited to the high fracture toughness induced by the high amount of the tetragonal (*t*) phase that is readily transformable to the monoclinic (*m*) symmetry [3] upon local stress, which induces a volumetric expansion ahead of propagating cracks [4]. Today, more translucent zirconias with reduced amount of stabilizer (i.e., 4YSZ and 5YSZ), and thus reduced amount of *t*-phase, find increasing clinical applicability, especially in the monolithic form, a trend ignited by the well-known chipping problem in veneered-zirconia systems [5, 6].

Conventionally, dental YZS ceramics are sintered for purpose of densification and grain growth in a process that takes up to 6–8 h from heating up to about 1500 °C, with a usual dwell time of 2 h and a relative slow cooling down phase. Such long sintering programs are conducted in conventional furnaces built using tubular heating elements that generate heat through resistive heating of ceramic conductive wires, such as silicon carbide (SiC, up to ~ 1625 °C) or molybdenum disilicide (MoSr_2_, up to ~ 1850 °C). This form of heat generation is slow compared to other technologies such as induction heating used in newer generations of sintering furnaces, which make use of an electromagnetic field induced to a copper coil in alternate current. That magnetic field creates eddy currents in the material that feels resistance toward flow, heating up by Joule heating. Induction furnaces are thus more efficient and can operate in high heating rates, and have recently entered the dental market for purpose of sintering zirconia ceramics, thus optimizing workflow in dental laboratories and enabling zirconia to be used also in chairside applications. With induction furnaces, sintering of dental zirconias following the so-called speed- and super-speed-sintering became approaches of highest interest, despite backed by limited experimental evidences. Initial evaluations seem to show that such protocols including fast heating and short dwell times can be used safely to sinter dental zirconias, with negligible adverse effects to density, translucency, and mechanical properties [7, 8]. Although the mechanical stability of speed-sintered zirconias has been covered in terms of force-at-fracture experiments and more standardized strength testing [9–12], the property of fracture toughness—of highest relevance than strength—has yet not been addressed in the context of speed-sintering.

Although induction furnaces constitute the state-of-the-art in sintering technology for dental zirconias, they also represent a significant financial investment that cannot be currently taken to be widespread, making the use of conventional furnaces still the standard practice. The purpose of this study was therefore to evaluate the feasibility of speed-sintering programs using a conventional furnace regarding the physical and mechanical properties, including Young’s modulus, biaxial flexural strength, and fracture toughness.

## Materials and methods

### Materials

The materials to be evaluated in this study were selected based on the degree of stabilization with Y_2_O_3_ so to encompass the range of conventional and translucent zirconias, i.e., 3 mol%, 4 mol%, or 5 mol% Y_2_O_3_. Two materials were selected per manufacturer; Table 1 summarizes their brand names, manufacturers, batches, and the quantification of Y_2_O_3_ using X-Ray Fluorescence Spectroscopy and the phase content using X-Ray Diffraction and Rietveld refinement considering the existence of two tetragonal phases [13]. The foregoing of using a cubic phase structure for the fit in the Rietveld refinement is based on the low sensitivity of XRD to perturbations on the anion sub-lattice, leading to the appearance of cubic-like peaks [14–17] despite forbidden in < 8 mol% Y_2_O_3_ compositions as demonstrated in selected-area diffraction in transmission electron microscopic studies revealing {112}-type reflections along the ❬111❭ zone axis [14, 15].

**Table 1:** Commercial materials analyzed in this study, their specified stabilizer content, manufacturers, batches, peak temperature, and dwell time of both conventional and speed-sintering programs, along with phase fractions

Material	Y_2_O_3_ [mol%]	Manufacturer	Batch (Lot Nr.)	Conventional sintering peak temperature [ °C]/dwell time [min]	Speed-sintering peak temperature [ °C]/dwell time [min]	*t* (Y-lean) [vol.%]^§^	*t”* (Y-rich) [vol.%]^§^
IPS e.max ZirCAD MO	3.08	Ivoclar-Vivadent AG, Liechtenstein	V38361	1500/120	1540/35	69.1 ± 0.8	30.9 ± 0.8
IPS e.max ZirCAD MT	4.28		W12059	1500/120	1540/35	53.5 ± 0.7	46.6 ± 0.7
Lava Plus	3.15	3 M Deutschland GmbH, Germany	3343987	1500/120	1540/35	67.7 ± 0.1	32.4 ± 0.1
Lava Esthetic	4.84		3515130	1500/120	1540/35	41.2 ± 2.3	58.8 ± 2.3
Cercon ht	3.12	Dentsply-Sirona Inc., Germany	18029331	1520/145	1540/35	69.0 ± 0.5	31.0 ± 0.5
Cercon xt	5.38		18031834	1520/145	1540/35	33.1 ± 0.3	66.9 ± 0.3
Katana ML	4.07	Kuraray Noritake Dental Inc., Japan	DTHYP	1500/120	1540/35	59.3 ± 0.7	40.7 ± 0.7
Katana STML	5.36		DLEEQ	1550/120	1540/35	36.4 ± 4.2	63.7 ± 4.2
Prettau	3.03	Zirkonzahn GmbH, Italy	ZB3235E	1600/120	1540/35	70.8 ± 0.1	29.2 ± 0.1
Prettau Anterior	5.40		ZB8068A	1500/120	1540/35	37.1 ± 4.8	62.9 ± 4.8

^**§**^Phase fraction quantified for the conventional sintering program only

**Fig. 1:**
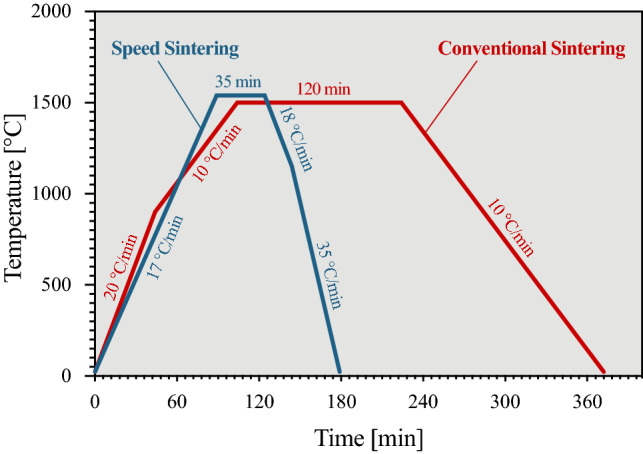
Example of a conventional (red) and the general speed-sintering (blue) programs utilized in this study

Here, an alternative sintering program “speed-sintering” was evaluated, and compared to the “conventional sintering” program in terms of physical and mechanical properties as reported in Refs. [13, 18]. Sintering was performed in a bottom-lift oven (Vita Zircomat 6000 M Speed, Vita Zahnfabrik) that allows flexible programming of the heating and cooling curves. The parameters for conventional sintering were maintained strictly according to the manufacturers’ recommendations, which varied slightly depending on material and manufacturer. The maximum sintering temperature varied between 1500 °C and 1600 °C, for a period between 120 and 145 min, with slow cooling taking place inside the oven overnight. Because most materials did not list in their instructions for use any alternative to their conventional sintering program, we devised a generalized speed-sintering program (see Table 1 and Fig. 1) (based on the available speed-sintering program detailed in the instructions for the Cercon materials) that would be feasible to use in typical sintering furnaces based on electrical resistive heating elements. It consisted of a ramp heating of 17 °C/min, holding time of 35 min at 1540 °C, and cooling rate of 18 °C/min until 1200 °C and 35 °C/min thereafter.

### Mechanical characterization

The Young’s modulus *E*, and the Poisson’s ratio *ν*, of the sintered samples were measured using Resonant Ultrasound Spectroscopy (RUS) as described in a previous study [19]. The bulk density *ρ* was determined geometrically. One specimen per material was used for each measurement due to previously determined high reproducibility [19], with three repetitions per specimen.

#### Measurement of the biaxial flexural strength

As-received partially sintered blanks (except for IPS e.max® MO, for which only B 40 L blocks were used) were sectioned with a band saw in smaller rectangular pieces and further cut in oversized (20%) dimensions under water irrigation using an automatic saw and diamond-coated copper discs. For each material, plates were sectioned to result in the dimensions 12 × 12 mm^2^ of thickness *t* = 1.2 mm after sintering, to be then tested in biaxial flexure. The surface of the specimens to be subjected to flexure was not further treated other than cleaned with water spray to remove the dust from sawing; no surface treatment was undergone after sintering.

To obtain the biaxial flexure strength, the Ball-on-Three-Balls (B3B) test configuration was used. Although originally designed for disc geometries [20], it was later adapted for rectangular plates and validated extensively [21, 22]. During the test, the maximum principal stress that develops on the tensile side of the specimen at fracture is taken as the strength at fracture, and is calculated as

1 $${\sigma }_{\mathrm{B}3\mathrm{B}}=\delta \frac{{F}_{\text{max}}}{{t}^{2}},$$

being *t* the thickness of the specimen, *F*_max_ the maximum force at fracture, and *δ* a function derived using finite element analysis, determined by two independent variables

2 $$\delta =f\left(\frac{t}{{R}_{a}},\nu \right)=0.323308+\frac{\left(1.30843+1.44301\nu \right)\times \left[1.78428-3.15347\left(t/{R}_{a}\right)+6.67919{\left(t/{R}_{a}\right)}^{2}-4.62603{\left(t/{R}_{a}\right)}^{3}\right]}{1+1.71955\left(t/{R}_{a}\right)},$$

where the support radius *R*_a_ = (2√3*R*_b_)/3 is formed by the three supporting balls of *R*_b_ = 4 mm and *ν* the Poisson´s ratio of the material.

At least 30 specimens were prepared for group, which were statistically treated using Weibull statistics according to the standard EN DIN 843–5 [23], and evaluated regarding the Weibull scale (*σ*_0_) and shape (*m*) parameters. Groups were considered statistically different the 90% confidence interval bands overlapped.

#### Measurement of the fracture toughness

The measurement of the fracture toughness was conducted using the Chevron Notched Beam (CNB) method according to the ‘Configuration A’ standardized in ASTM C 1421, having a cross section height *W* × width *B* of 4 mm × 3 mm (a geometry also standardized in ISO 24370 and EN 14425–3), having beam length *L* of 25 mm to be measured in four-point bending with outer and inner spans of 20 mm and 10 mm, respectively. For that, blanks of partially sintered material (for IPS e.max® CAD MO B 40 L blocks) were used as received from the manufacturers. Beams were sawed from the partially sintered blanks/block under water lubrication using an automatic cutting saw (Bühler 5000) and a diamond-coated copper disc and cut in oversized dimensions to account for ~ 20% linear shrinkage. The notch at the midspan of the beams followed the notch dimensional ratios recommended in the aforementioned standards and was produced at the white-body stage (prior to sintering) by means of successive cuts using a rotating 0.15 mm-thick diamond disc. Up to 12 specimens were produced per material for each experimental group accounting for the eventuality of invalid tests.

**Fig. 2:**
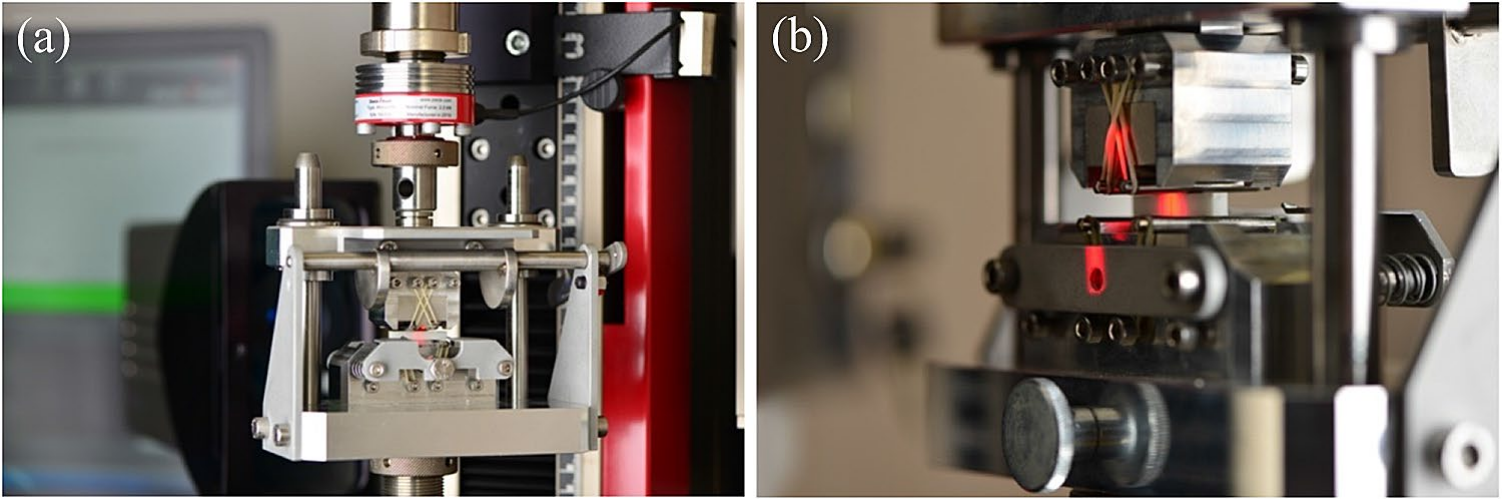
Fully articulated jig used for testing in four-point bending, coupled with a laser-unit for speckle image correlation to track the specimen deflection and the presence of subcritical crack growth before fracture

Notched specimens were sintered with the notch tip directed upwards in a tray containing zirconia balls, with all specimens per group sintered together in the same cycle. In the occasion of slight sintering deformations, specimens were made plane-parallel in a grinding machine under water irrigation. The notch dimensions on the lateral sides of the final specimens were measured under a stereomicroscope coupled with a digital camera and accompanying software. Before testing, the specimens were dried in an oven at 150 °C together with a silicon oil bath, into which the specimens were immersed after 3 h of drying. This was meant to prevent any potential water-assisted stress corrosion crack growth at the crack that pops at the tip of the triangular notch during testing, which could induce and influence the obtained *K*_Ic_-values [24]. Specimens coated with silicon oil were tested at a loading rate of 0.005 mm/s (to induce a pop-in crack) in a custom testing jig [25] (see Fig. 2), with load-line displacement controlled by an imaging system (LaserXtens, Zwick/Roell) based on the image digital correlation approach for accurate detection of the stable crack growth at the tip of the notch before instability. The *K*_Ic_ was then calculated from the maximum force at fracture *F*_max_ [26]

3 $${K}_{\mathrm{Ic}}=\frac{{F}_{\mathrm{max}}\left({S}_{\mathrm{o}}-{S}_{\mathrm{i}}\right)}{B{W}^{3/2}}\bullet \frac{{Y}_{\mathrm{min}}^{*}}{\sqrt{{10}^{3}}},$$

being *S*_o_ the outer span length and *S*_i_ the inner span length, and for four-point bending with the configuration A

4 $${Y}_{\mathrm{min}, 4\mathrm{PB}}^{*}=\frac{0.3874-3.0919\left({l}_{0}/W\right)+4.2017\left({l}_{1}/W\right)-2.3127{\left({l}_{1}/W\right)}^{2}+0.6379{\left({l}_{1}/W\right)}^{3}}{1-2.9686\left({l}_{0}/W\right)+3.5056{\left({l}_{0}/W\right)}^{2}-2.1374{\left({l}_{0}/W\right)}^{3}+0.0130\left({l}_{1}/W\right)},$$

where *l*_0_ is the distance between the bottom edge of the beam and the tip of the Chevron notch, and *l*_1_ is an arithmetic mean of the notched segments on the sides of the beam. The ratios *l*_0_/W and *l*_1_/*W* were kept within the ranges 0.175 < *l*_0_/W < 0.225 and 0.95 < *l*_1_/W < 1 for configuration A, to minimize the error to a maximum of 1%. The *l*_0_ was measured after fracture in a stereomicroscope coupled with a digital camera and accompanying software. Specimens showing load–deformation curves diverging from those depicted in the aforementioned standards (absence of stable crack propagation before instability) were regarded as invalid tests and not included in the analysis. The aforementioned standards define a sample number of 5 valid specimens as sufficient for evaluation; we made sure to obtain 9–10 valid specimens for each material. Our CNB testing procedures have been recently validated using a Standard Reference Material [27].

For comparison within the same material between the two sintering programs, multiple unpaired Student’s t test were conducted; among materials within the same sintering program, ANOVA test followed by a Tukey’s post hoc test was conducted. A level of significance *α* = 0.05 was defined.

## Results

**Fig. 3:**
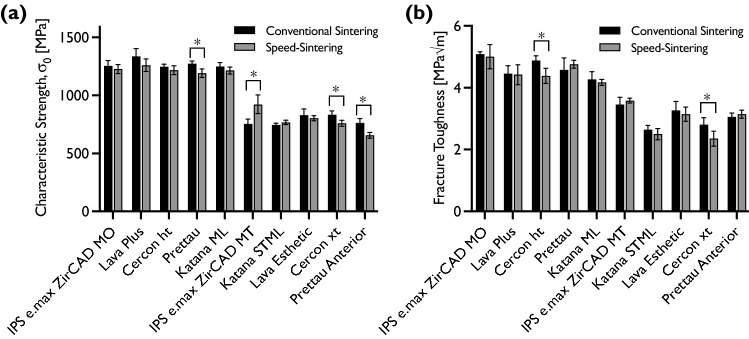
Plots of the characteristic strength (90% C.I.) and fracture toughness (S.D.) for the two sintering programs. Asterisk represents significant differences within groups

The Young’s modulus, density, and Poisson’s ratio are listed in Table 2 and showed no statistical different between sintering programs. The results of the biaxial flexural strength testing, treated using two-parameter Weibull statistics, are detailed in Table 3 in terms of the scale σ_0_ and shape (*m*) parameters of the distribution. The results of the fracture toughness are also included in Table 3. In Fig. 3, the results of the characteristic strength and fracture toughness are summarized side-by-side.

**Table 2:** Results of the resonant ultrasound spectroscopy measurement of the Young’s modulus E, the Poisson’s ratio *ν*, and the density *ρ*

Material	Conventional sintering			Speed-sintering		
	*E* [GPa]	*ν*	*ρ* [g/cm^3^]	*E* [GPa]	*ν*	*ρ* [g/cm^3^]
IPS e.max ZirCAD MO	212.7 ± 1.1	0.315 ± 0.010	5.976 ± 0.039	212.7 ± 1.1	0.314 ± 0.010	6.037 ± 0.021
Lava Plus	214.3 ± 1.1	0.314 ± 0.010	6.053 ± 0.014	214.5 ± 1.0	0.314 ± 0.010	6.091 ± 0.013
Cercon ht	214.2 ± 1.1	0.317 ± 0.010	6.069 ± 0.017	213.8 ± 1.1	0.316 ± 0.010	6.068 ± 0.014
Prettau	214.1 ± 1.1	0.313 ± 0.010	6.080 ± 0.016	214.3 ± 1.1	0.317 ± 0.010	6.075 ± 0.045
Katana ML	217.4 ± 1.1	0.315 ± 0.010	6.041 ± 0.016	213.7 ± 1.1	0.310 ± 0.010	6.052 ± 0.033
IPS e.max ZirCAD MT	214.7 ± 1.3	0.314 ± 0.010	6.035 ± 0.018	215.2 ± 1.1	0.314 ± 0.010	6.057 ± 0.018
Katana STML	214.5 ± 1.1	0.314 ± 0.010	5.988 ± 0.037	213.3 ± 1.1	0.314 ± 0.010	6.049 ± 0.018
Lava Esthetic	215.3 ± 1.1	0.314 ± 0.010	6.053 ± 0.018	215.0 ± 1.1	0.313 ± 0.010	6.066 ± 0.018
Cercon xt	216.2 ± 1.1	0.315 ± 0.010	6.0256 ± 0.008	216.1 ± 1.1	0.316 ± 0.010	6.003 ± 0.024
Prettau Anterior	216.9 ± 1.1	0.312 ± 0.010	6.016 ± 0.014	214.9 ± 1.1	0.302 ± 0.010	5.972 ± 0.057

**Table 3:** Results of the mechanical testing of biaxial flexural strength (in terms of Weibull modulus *m* and characteristic strength *σ*_0_, with corresponding 90% confidence intervals) and fracture toughness *K*_Ic_ (S.D.)

Material	Conventional sintering			Speed-sintering		
	*K*_Ic_ [MPa√m]	*σ*_0_ [MPa]	*m*	*K*_Ic_ [MPa√m]	*σ*_0_ [MPa]	*m*
IPS e.max ZirCAD MO	5.08 ± 0.08^a^	1253.9 [1209–1300]^ab^	9.0 [7.1–11.4]^bc^	5.00 ± 0.39 ^a^	1227.2 [1189–1266]^a^	9.7 [7.7–12.4]^a^
Lava Plus	4.45 ± 0.26^bc^	1336.7 [1272–1404]^a^	6.6 [5.2–8.4]^cd^	4.42 ± 0.32 ^c^	1259.7 [1206–1315]^a^	7.1 [5.7–9.1]^b^
Cercon ht	4.87 ± 0.16^a§^	1246.2 [1224–1268]^b^	18.6 [14.7–23.5]^a*^	4.38 ± 0.24 ^c§^	1216.9 [1180–1255]^a^	10.1 [7.9–12.8]^a*^
Prettau	4.57 ± 0.39^bc^	1273.2 [1249–1297]^ab#^	17.0 [13.4–21.5]^a*^	4.76 ± 0.13 ^b^	1191.2 [1155–1228]^a#^	10.2 [8.1–12.9]^a*^
Katana ML	4.27 ± 0.25^c^	1248.9 [1216–1283]^ab^	12.1 [9.6–15.4]^ab^	4.17 ± 0.10 ^d^	1215.1 [1185–1245]^a^	12.7 [10.1–16.2]^a^
IPS e.max ZirCAD MT	3.45 ± 0.24^d^	754.2 [714–797]^de#^	5.9 [4.6–7.4]^cd*^	3.58 ± 0.08 ^e^	920.7 [844–1004]^b#^	3.8 [2.8–4.5]^c*^
Katana STML	2.64 ± 0.14^ g^	744.1 [727–761]^e^	13.9 [11.0–17.5]^ab^	2.50 ± 0.18 ^g^	767.8 [748–787]^c^	12.3 [9.7–15.5]^a^
Lava Esthetic	3.26 ± 0.3^d^	829.9 [779–883]^cd^	5.2 [4.1–6.6]^d*^	3.14 ± 0.23 ^f^	803.7 [782–825]^c^	11.4 [9.0–14.5]^a*^
Cercon xt	2.80 ± 0.23^ fg§^	832.6 [800–866]^c#^	8.2 [6.5–10.3]^cd^	2.35 ± 0.24 ^g^^§^	759.8 [734–785]^c#^	9.3 [7.3–11.8]^a^
Prettau Anterior	3.05 ± 0.13^f^	761.5 [724–800]^cde#^	6.5 [5.1–8.2]^cd^	3.14 ± 0.13 ^f^	656.8 [631–682]^d#^	8.0 [6.3–10.1]^a^

For *m* and *σ*_0_, statistical significance was established by the overlapping of the confidence intervals. For *K*_Ic_, multiple Student’s *t *tests were performed between sintering protocols, and ANOVA followed by Tukey’s post hoc test was performed within sintering protocols, all at a significance level *α* = 0.05

Same superscript letters (a–g) within columns belong to same statistical subset (Tukey’s test)

Same superscript symbols (§, #, *) within rows distinguish between different statistical subsets (Student’s *t *test)

Although there were significant differences in characteristic strength for some of the materials between the two sintering programs, speed-sintering did not always lead to a reduction of the strength, with the material IPS e.max ZirCAD MT showing a significant increase in strength after speed-sintering. Also, differences were not restricted to only one material type (amount of stabilizer), but affected 3, 4, and 5 mol% Y_2_O_3_-stabilized materials. Regarding fracture toughness, statistically significant differences were only observed for the two Cercon materials, where speed-sintering reduced the fracture toughness. Otherwise, for all other materials, the fracture toughness was statistically comparable between the sintering programs.

## Discussion

Regarding the statistical treatment of strength data, *m* is a representation of the distribution of critical defect sizes responsible for triggering the fracture in the sampled effective surface/volume, which is dependent on the specimen size and loading configuration. In the context of sintering, significant changes in *m* suggest a different dynamic in the densification of the white-body, related not to the heat distribution once both heating rates were very similar, but to the dwell time. Belli and Lohbauer [18] clearly showed that in dental zirconias, the defect distribution in the fully sintered piece is inherited from the defect distribution in the white-body. Passed down to the sintered analogs, the morphology of critical defects in the white-body constitute junction vertices between spherical spray-dried granulates that failed to reach intimate contact during pressing. It is conjectured that during uniaxial compaction, the stiff binder at the outer granule shell becomes trapped at the vertices, assuming polyhedral shapes that further oppose compaction [28]; the burn-out of the binder concomitant to the shrinkage of the granules toward their center of mass leaves behind empty spaces at the junctions that cannot be filled by mass transport [29]. The morphology of such defects is a three-dimensional “crowfoot” with “spikes” that are invariably unfavorably oriented in relation to the direction of tension. The parameter *m* was reduced significantly for three materials, namely Cercon ht, Prettau, and IPS e.max ZirCAD MT, while increasing for Lava Esthetic. In the speed-sintered specimens, apart from IPS e.max ZirCAD MT, all material showed a Weibull modulus between 7 and 13, showing less variability than under conventional sintering conditions (*m* between 6 and 19). Another sign of the sintering strategy having an effect on how sintering defects develop was a change in *σ*_0_, which represents the strength at a 63.2% failure probability as a reflection of the scale of the sizes of critical defects. That was seen in Prettau, Prettau Anterior, and Cercon xt leading to a significant reduction of *σ*_0_, with the opposite effect in IPS e.max ZirCAD MT. A decrease in *σ*_0_ might imply that defects remained larger compared to when the dwell time is increased from 35 min to 2 h. Possibly, an increased dwell time favors sintering to extend and close existing voids in the white-body, especially at the edges of defects, where the faces between pressed granules are in closer contact. The effect, however, does not seem to be systematic or dependent on stabilizer content, but be rather material dependent. Comparisons to the literature are not always straightforward in respect to speed-sintering, since time, temperatures, furnaces, and heating principles (e.g., induction, plasma, microwave, etc.) are particular for each study. Nonetheless, some patterns can be recognized, namely, most strength experiments report no significant differences to the conventional sintering [7, 8, 11, 30, 31], with few observing even some improvement [32]. Kaizer et al. [33] though showed that speed and super-speed-sintering lead to higher amounts of pitting during contact sliding wear compared to conventional sintering, pointing to densification issues. Other studies describe that speed-sintering led to a change in grain size distribution [7, 8], with speed-sintering inducing larger fraction of fine grains, and a decrease in medium grains [8]. In terms of phase fractions, sintering protocol seems to result in changes in the proportion of tetragonal and cubic grains, but this phenomenon does not seem to follow a certain pattern, but also be material dependent [7, 8].

Microstructural aspects, specifically grain size, have shown to affect the distribution of the stabilizer within the grains, in turn determining the tetragonal to monoclinic transformability, the property which confers the highest gain in toughening in yttria-stabilized zirconias [3]. Actually, using the phase content of the materials evaluated here sintered conventionally, including a 2 mol% Y_2_O_3_-stabilized zirconia, we established a power-law relationship between *K*_Ic_ and the volume content of the *t*-phase [13]. That led to an inverse power-law relationship between *K*_Ic_ and yttria content of the form *K*_Ic_(*x*Y_2_O_3_) = *K*_I,0_ *e*^−λ*x*^ + *K*_Ic,cubic_, with λ = -1.102 mol%^−1^, challenging the classic relationship of Lange [34] and agreeing rather to the trend seen by Masaki [35]. With speed-sintering, that relationship is not disturbed, suggesting that the main factors affecting the fracture toughness, namely grain size distribution and *t*-phase content, remained mostly preserved with the decrease in dwell time. Two exceptions must be noted here, namely, the fracture toughness of the materials Cercon ht and Cercon xt, both from the manufacturer Dentsply-Sirona, were the only ones that showed a significant decrease (10% and 16%, respectively) resulting from speed-sintering. Interestingly, the speed-sintering program utilized here corresponded exactly to the heating rate, maximum temperature, and dwell time recommended by Dentsply-Sirona. For both products though, the conventional sintering program had the longest dwell time of all products, which might have actually contributed to an increased *K*_Ic_-value for the conventionally sintered specimens. When compared to the materials having the same composition (Lava Plus and Prettau; IPS e.max ZirCAD MO has a Al_2_O_3_ of 0.3 mol%, significantly higher than all other materials), Cercon ht shows the highest *K*_Ic_-value when conventionally sintered, and still statistically similar to Lava Plus despite its drop after speed-sintering. For Cercon xt, its *K*_Ic_-value despite now statistically lower than Prettau Anterior remained statistically similar to Katana STML. In that view, the drop in *K*_Ic_-values for both materials after speed-sintering might be relativized, once still in the range of other materials for comparable compositions.

The comparison of conventional vs. speed-sintering protocols was here performed in the same furnace based on electrical resistive heating elements, and could be supplemented by future research evaluating the use of induction furnaces. Additionally, even though values of strength and fracture toughness can be indicative of the phase composition in dental zirconias, as shown in Refs. [13] and [18], further evaluations on microstructural parameters (grain size and distribution) and phase content using scanning electron microscopy and X-ray diffraction, respectively, would be warranted for additional insights on more specific effects of speed-sintering relative to conventional sintering programs.

## Conclusions

From the results of the present study, we can conclude that sintering dental zirconias (3-5YSZ) using a speed-sintering protocol in a conventional furnace does not significantly compromise their mechanical properties and could be adopted safely, in detriment of the longer, more energy-consuming conventional sintering protocol.

## Data Availability

Data will be made available at request.

## References

[1] Miura S, Yamauchi S, Kasahara S, Katsuda Y, Fujisawa M, Egusa H (2021). Clinical evaluation of monolithic zirconia crowns: a failure analysis of clinically obtained cases from a 3.5-year study. J Prosthodont Res.

[2] Belli R, Petschelt A, Hofner B, Hajto J, Scherrer SS, Lohbauer U (2016). Fracture rates and lifetime estimations of CAD/CAM all-ceramic restorations. J Dent Res.

[3] Ruhle M, Evans AG (1989). High toughness ceramics and ceramic composites. Prog Mater Sci Prog Mater Sci.

[4] Chevalier J, Gremillard L, Virkar AV, Clarke DR (2009). The tetragonal-monoclinic transformation in zirconia: lessons learned and future trends. J Am Ceram Soc.

[5] Sailer I, Pjetursson BE, Zwahlen M, Hammerle CHF (2007). A systematic review of the survival and complication rates of all-ceramic and metal-ceramic reconstructions after an observation period of at least 3 years Part II: fixed dental prostheses. Clin Oral Implan Res Clin Oral Implan Res.

[6] Belli R, Frankenberger R, Appelt A, Schmitt J, Baratieri LN, Greil P (2013). Thermal-induced residual stresses affect the lifetime of zirconia-veneer crowns. Dent Mater.

[7] Cokic SM, Vleugels J, Van Meerbeek B, Camargo B, Willems E, Li M (2020). Mechanical properties, aging stability and translucency of speed-sintered zirconia for chairside restorations. Dent Mater.

[8] Liu H, Inokoshi M, Nozaki K, Shimizubata M, Nakai H, Cho Too TD (2022). Influence of high-speed sintering protocols on translucency, mechanical properties, microstructure, crystallography, and low-temperature degradation of highly translucent zirconia. Dent Mater.

[9] Zimmermann M, Ender A, Mehl A (2020). Influence of CAD/CAM fabrication and sintering procedures on the fracture load of full-contour monolithic Zirconia crowns as a function of material thickness. Oper Dent.

[10] Too TDC, Inokoshi M, Nozaki K, Shimizubata M, Nakai H, Liu H (2021). Influence of sintering conditions on translucency, biaxial flexural strength, microstructure, and low-temperature degradation of highly translucent dental zirconia. Dent Mater J.

[11] Lawson NC, Maharishi A (2020). Strength and translucency of zirconia after high-speed sintering. J Esthet Restor Dent.

[12] Jansen JU, Lumkemann N, Letz I, Pfefferle R, Sener B, Stawarczyk B (2019). Impact of high-speed sintering on translucency, phase content, grain sizes, and flexural strength of 3Y-TZP and 4Y-TZP zirconia materials. J Prosthet Dent.

[13] Belli R, Hurle K, Schurrlein J, Petschelt A, Werbach K, Peterlik H (2021). Relationships between fracture toughness, Y2O3 fraction and phases content in modern dental Yttria-doped zirconias. J Eur Ceram Soc.

[14] Krogstad JA, Lepple M, Gao Y, Lipkin DM, Levi CG (2011). Effect of Yttria content on the Zirconia unit cell parameters. J Am Ceram Soc.

[15] Krogstad JA, Leckie RM, Kramer S, Cairney JM, Lipkin DM, Johnson CA (2013). Phase evolution upon aging of air plasma Sprayed t'-Zirconia coatings: II-microstructure evolution. J Am Ceram Soc.

[16] Lipkin DM, Krogstad JA, Gao Y, Johnson CA, Nelson WA, Levi CG (2013). Phase evolution upon aging of air-plasma Sprayed t'-Zirconia coatings: Isynchrotron X-Ray diffraction. J Am Ceram Soc.

[17] Krogstad JA, Gao Y, Bai JM, Wang J, Lipkin DM, Levi CG (2015). In situ diffraction study of the high-temperature decomposition of t '-Zirconia. J Am Ceram Soc.

[18] Belli R, Lohbauer U (2021). The breakdown of the Weibull behavior in dental zirconias. J Am Ceram Soc.

[19] Belli R, Wendler M, de Ligny D, Cicconi MR, Petschelt A, Peterlik H (2017). Chairside CAD/CAM materials Part 1: measurement of elastic constants and microstructural characterization. Dent Mater.

[20] Börger A, Supancic P, Danzer R (2002). The ball on three balls test for strength testing of brittle discs: stress distribution in the disc. J Eur Ceram Soc.

[21] Danzer R, Supancic P, Harrer W (2006). Biaxial tensile strength test for brittle rectangular plates. J Ceram Soc Jpn.

[22] Harrer W, Danzer R, Supancic P, Lube T. The ball on three balls test: strength testing of specimens of different sizes and geometries. Proc ECerS Conf 2007; 1271–5.

[23] DIN EN 843–5. Mechanical testing of monolitic ceramics at room temperature. Part 5: Statistical treatment. DIN Deutsches Institut für Normung e.V., 1997

[24] Krautgasser C, Chlup Z, Supancic P, Danzer R, Berrnejo R (2016). Influence of subcritical crack growth on the determination of fracture toughness in brittle materials. J Eur Ceram Soc.

[25] Belli R, Wendler M, Zorzin JI, Lohbauer U (2018). Practical and theoretical considerations on the fracture toughness testing of dental restorative materials. Dent Mater.

[26] ASTM C 1421. Standard test methods for determination of fracture toughness of advances ceramics at ambient temperature. ASTM International; 2010.

[27] Belli R, Wendler M, Petschelt A, Lube T, Lohbauer U (2018). Fracture toughness testing of biomedical ceramic-based materials using beams, plates and discs. J Eur Ceram Soc.

[28] Boursier A, d'Esdra GG, Lintingre E, Fretigny C, Lequeux F, Talini L (2020). Cold compression of ceramic spray-dried granules: Role of the spatial distribution of the binder. Ceram Int.

[29] Hondo T, Yasuda K, Wakai F, Tanaka S (2018). Influence of binder layer of spray-dried granules on occurrence and evolution of coarse defects in alumina ceramics during sintering. J Eur Ceram Soc.

[30] Jerman E, Wiedenmann F, Eichberger M, Reichert A, Stawarczyk B (2020). Effect of high-speed sintering on the flexural strength of hydrothermal and thermo-mechanically aged zirconia materials. Dent Mater.

[31] Yang C-C, Ding S-J, Lin T-H, Yan M (2020). Mechanical and optical properties evaluation of rapid sintered dental zirconia. Ceram Int.

[32] Ersoy NM, Aydogdu HM, Degirmenci BU, Cokuk N, Sevimay M (2015). The effects of sintering temperature and duration on the flexural strength and grain size of zirconia. Acta Biomater Odontol Scand.

[33] Kaizer MR, Gierthmuehlen PC, dos Santos MBF, Cava SS, Zhang Y (2017). Speed sintering translucent zirconia for chairside one-visit dental restorations: Optical, mechanical, and wear characteristics. Ceram Int.

[34] Lange FF (1982). Transformation toughening 3 experimental-observations in the Zro_2_-Y2o_3_ system. J Mater Sci.

[35] Masaki T (1986). Mechanical-properties of toughened Zro_2_-Y2o_3_ Ceramics. J Am Ceram Soc.

